# Papers Presented

**Published:** 1867-06

**Authors:** 


PAPERS PRESENTED.

The Secretary presented the following named papers, which were referred to appropriate sections:

1. Observations on the Diseases of the Heart, as seen in Military Service from 1861 to 1865, by M. K. Taylor.
2. Novel Case of Lithotomy, by Dr. Edward Whinney, of Iowa.
3. Report on Ligature of the Subclavian Artery, by Dr. Parker.
4. A communication from Chicago, informing the Association that during 1866, two members of the profession, named, had been expelled from their local Medical Society, for violation of the Code of Ethics.
5. A communication proposing that no person who is not a member and supporter of a local Medical Society, where such a one exists, shall be eligible to membership in the American Medical Association.

This being virtually an amendment to the Constitution, it was laid over for one year.

